# A prognostic exosome-related LncRNA risk model correlates with the immune microenvironment in liver cancer

**DOI:** 10.3389/fgene.2022.965329

**Published:** 2022-08-23

**Authors:** Duntao Su, Zeyu Zhang, Zhijie Xu, Fada Xia, Yuanliang Yan

**Affiliations:** ^1^ Department of General Surgery, Xiangya Hospital, Central South University, Changsha, Hunan, China; ^2^ Department of Clinical Laboratory, Xiangya Hospital, Central South University, Changsha, China; ^3^ Department of Pathology, Xiangya Changde Hospital, Changde, Hunan, China; ^4^ National Clinical Research Center for Geriatric Disorders, Xiangya Hospital, Central South University, Changsha, Hunan, China

**Keywords:** liver cancer, exosomes, lncRNAs, prognostic signature, tumor immune microenvironment

## Abstract

**Background:** Emerging studies have shown the important roles of long noncoding RNAs (lncRNAs) in the occurrence and development of liver cancer. However, the exosome-related lncRNA signature in liver cancer remains to be clarified.

**Methods:** We obtained 371 tumor specimens and 50 normal tissues from the TCGA database. These samples were randomly divided into the training queue and verification queue. The exosome-related lncRNA risk model was verified by correlation analysis, Lasso regression analysis, and Cox regression analysis. The differences in the immune microenvironment in the two risk groups were obtained by analyzing the infiltration of different immune cells.

**Results:** Five exosome-related lncRNAs associated (MKLN1-AS, TMCC1-AS1, AL031985.3, LINC01138, AC099850.3) with a poor prognosis were identified and used to construct the signature. Receiver operating curve (ROC) and survival curves were used to confirm the predictive ability of this signature. Based on multivariate regression analysis in the training cohort (HR: 3.033, 95% CI: 1.762–5.220) and validation cohort (HR: 1.998, 95% CI: 1.065–3.751), the risk score was found to be an independent risk factor for patient prognosis. Subsequently, a nomogram was constructed to predict the 1-, 3-, 5-years survival rates of liver cancer patients. Moreover, this signature was also related to overexpressed immune checkpoints (PD-1, B7-H3, VSIR, PD-L1, LAG3, TIGIT and CTLA4).

**Conclusion:** Our study showed that exosome-related lncRNAs and the corresponding nomogram could be used as a better index to predict the outcome and immune regulation of liver cancer patients. This signature might provide a new idea for the immunotherapy of liver cancer in the future.

## Background

Primary liver cancer is the sixth most diagnosed cancer and the third leading cause of death globally. Its incidence has been rising in recent years and it includes hepatocellular carcinoma (HCC) and intrahepatic cholangiocarcinoma (Sung et al., 2021). The treatment methods for liver cancer mainly include surgery, chemotherapy, and radiotherapy (Viveiros et al., 2019; Li and Chen, 2022). Although there have been breakthroughs in therapeutic strategies in recent years, the patient prognosis remains poor (Li et al., 2020). Therefore, we should classify liver cancer patients according to their specific conditions and risk score, which is more conducive to individualized precision medical treatment and improves the patient prognosis. A powerful predictor is needed to screen liver cancer patients to improve the effects of existing immunotherapy and to predict and improve the outcomes of patients.

Exosomes are microcapsules with an extracellular size of 30–100 nm released by various cells, including tumor cells and immune cells (Thakur et al., 2021). Exosomes have been proven to be related to various human diseases, including liver cancer. The latest studies showed that exosomes from HCC cells could provide favorable conditions for the proliferation, metastasis and drug resistance of HCC cells (Shen et al., 2020). In addition, liver cancer cell-derived exosomes can also be a new biomarker for the early diagnosis of HCC (Lee et al., 2019).

Long noncoding RNAs (lncRNAs) are RNAs whose length is not less than 200 nt that do not encode a protein but can regulate the level of gene expression. An increasing number of studies have shown that lncRNAs have a significant effect on the development and immune response of tumors (Zhang et al., 2021). LncRNAs are closely to liver cancer occurrence, development and prognosis (Ma et al., 2020; Xu et al., 2021). However, few studies on exosome-lncRNAs interactions in liver cancer have been reported. More importantly, constructing an exosome-related lncRNA signature could help us predict the outcome and therapeutic response of patients with liver cancer.

The aim of our study was to construct a predictive signature based on exosome-related lncRNAs. The signature could be used to explore the roles of exosome-lncRNAs in the regulation of the immune microenvironment and prognosis in liver cancer patients.

## Materials and methods

### Datasets and clinicopathological information acquisition

The liver cancer datasets containing RNA sequences were derived from TCGA (http://cancergenome.nih.gov/). Two patients were excluded because they did not have enough clinicopathological information. A total of 371 tumor and 50 normal tissue samples were included in our study.

### Identification of exosome-related lncRNAs

According to previous studies, a total of 120 exosome related genes are shown in Supplementary Table S1 (Wang et al., 2021; Xu et al., 2022). Pearson correlation was used to explore the relationship between lncRNAs and exosome-related genes. The screening criteria for Pearson correlations were coefficient >0.5 and *p* < 0.001.

### Development and validation of the prognostic exosome-related LncRNA signature

We randomly divided the samples into a training queue and verification queue at a ratio of 2:1. We first used the training cohort to construct a prognosis-related exosome-related lncRNA risk model, while the verification queue was used as a validation cohort. Univariate and multivariate Cox regression analyses were used to confirm the prognostic factors in HCC patients. Subsequently, least absolute shrinkage and selection operator (LASSO) regression was used to construct the exosome-related lncRNA signature. We got 5 exosome-related lncRNAs to construct the model through the “glmnet” R package. In this model, we used the following formula to calculate the patient’s risk score: risk score = expression of lncRNA1 × b1lncRNA1 + expression of lncRNA2×b2lncRNA2 +... expression of lncRNA × bnlncRNAn. We divided the two cohorts into high-risk and low-risk groups according to the median risk score. To compare the overall survival difference between the low- and highrisk groups, we used the “survminer” R package. To investigate the predictive ability of the prognostic model over time, we employed the “TimeROC” R package to show the time-dependent ROC curve. Kaplan‒Meier (K-M) survival analysis and receiver operating characteristic curve (ROC) were further used to verify the prognostic effect of the signature. A nomogram containing clinicopathological information was constructed based on the multivariate regression analysis. We used the “rms” R package to build the nomogram to predict the 1-, 3- and 5-years survival rates of HCC patients.

### The mRNA‒lncRNA coexpression network

To better explain the relationship between exosome-related genes and exosome-related lncRNAs, we constructed a coexpression network to explain their potential relationship. A Sankey diagram further clarified the relationships among the lncRNAs, mRNAs, and risk types.

### Gene set enrichment analysis and subsequent functional enrichment analyses

Differentially expressed genes between the groups were identified by the “limma” package with cutoff criteria of false discovery rate <0.05 and |log_2_foldchange| > 1. Subsequently, we uploaded differentially expressed genes to GSEA (http://www.broadinstitute.org/gsea) (Subramanian et al., 2005) for gene enrichment analysis. CIBERSORT (Barbie et al., 2009) was also used to illustrate the roles of the signature in the regulation of tumor-infiltrating immune cells in HCC tissues.

### Statistical analyses

We used R version 3.30 and the R package for all statistical calculations. We used the t-test or Wilcoxon test for group comparisons. The “Rtsne” R package was used to analyze t-SNE. Univariate and multivariate Cox regression analysis was run by the “survival” R package. One-way analysis of variance (ANOVA) or Welch’s ANOVA was used to compare the two samples. Univariate and multivariate regression analyses evaluated the model’s predictive value. Generally, *p* < 0.05 was considered statistically significant.

## Results

### Screening of exosome-related prognostic lncRNAs in liver cancer

First, we randomly divided the 371 TCGA-HCC patients into a training cohort of 247 and a validation cohort of 124. Table 1 shows the clinicopathological information characteristics of the two cohorts. There was no significant difference in any clinicopathological features.

**TABLE 1:** The characteristics of liver cancer patients in the training cohort and validation cohort.

Characteristic	Training cohort (*n* = 247)	Validation cohort (*n* = 124)	*p*-value
Age, median (IQR)	61 (52, 68)	60.5 (51, 70)	0.896
BMI, median (IQR)	24.26 (21.89, 28.24)	25.16 (20.94, 29.75)	0.527
Gender, n (%)			0.629
Female	78 (21%)	43 (11.6%)	
Male	169 (45.6%)	81 (21.8%)	
Family cancer history, n (%)			1.000
No	136 (42.5%)	72 (22.5%)	
Yes	74 (23.1%)	38 (11.9%)	
Race, n (%)			1.000
American indian or alaska native	2 (0.6%)	0 (0%)	
Asian	105 (29.1%)	53 (14.7%)	
Black or african American	11 (3%)	6 (1.7%)	
White	123 (34.1%)	61 (16.9%)	
Alcohol consumption, n (%)			0.076
No	149 (42.3%)	86 (24.4%)	
Yes	86 (24.4%)	31 (8.8%)	
Hepatitis B, n (%)			0.817
No	167 (47.4%)	81 (23%)	
Yes	68 (19.3%)	36 (10.2%)	
Hepatitis C, n (%)			0.372
No	201 (57.1%)	95 (27%)	
Yes	34 (9.7%)	22 (6.2%)	
T stage, n (%)			0.419
T1	116 (31.4%)	65 (17.6%)	
T2	62 (16.8%)	32 (8.7%)	
T3	57 (15.4%)	23 (6.2%)	
T4	10 (2.7%)	3 (0.8%)	
TX	0 (0%)	1 (0.3%)	
N stage, n (%)			0.393
N0	169 (45.7%)	83 (22.4%)	
N1	4 (1.1%)	0 (0%)	
NX	73 (19.7%)	41 (11.1%)	
M stage, n (%)			0.961
M0	176 (47.4%)	90 (24.3%)	
M1	3 (0.8%)	1 (0.3%)	
MX	68 (18.3%)	33 (8.9%)	
Pathologic stage, n (%)			0.314
Stage I	108 (31.1%)	63 (18.2%)	
Stage II	56 (16.1%)	30 (8.6%)	
Stage III	63 (18.2%)	22 (6.3%)	
Stage IV	4 (1.2%)	1 (0.3%)	
Neoplasm histologic grade, n (%)			0.631
G1	36 (9.8%)	19 (5.2%)	
G2	113 (30.9%)	64 (17.5%)	
G3	86 (23.5%)	36 (9.8%)	
G4	9 (2.5%)	3 (0.8%)	
Child-pugh classification grade, n (%)			0.370
A	142 (59.4%)	75 (31.4%)	
B	15 (6.3%)	6 (2.5%)	
C	0 (0%)	1 (0.4%)	
Microvascular invasion, n (%)			0.967
Yes	72 (22.9%)	37 (11.7%)	
None	138 (43.8%)	68 (21.6%)	

**FIGURE 1:**
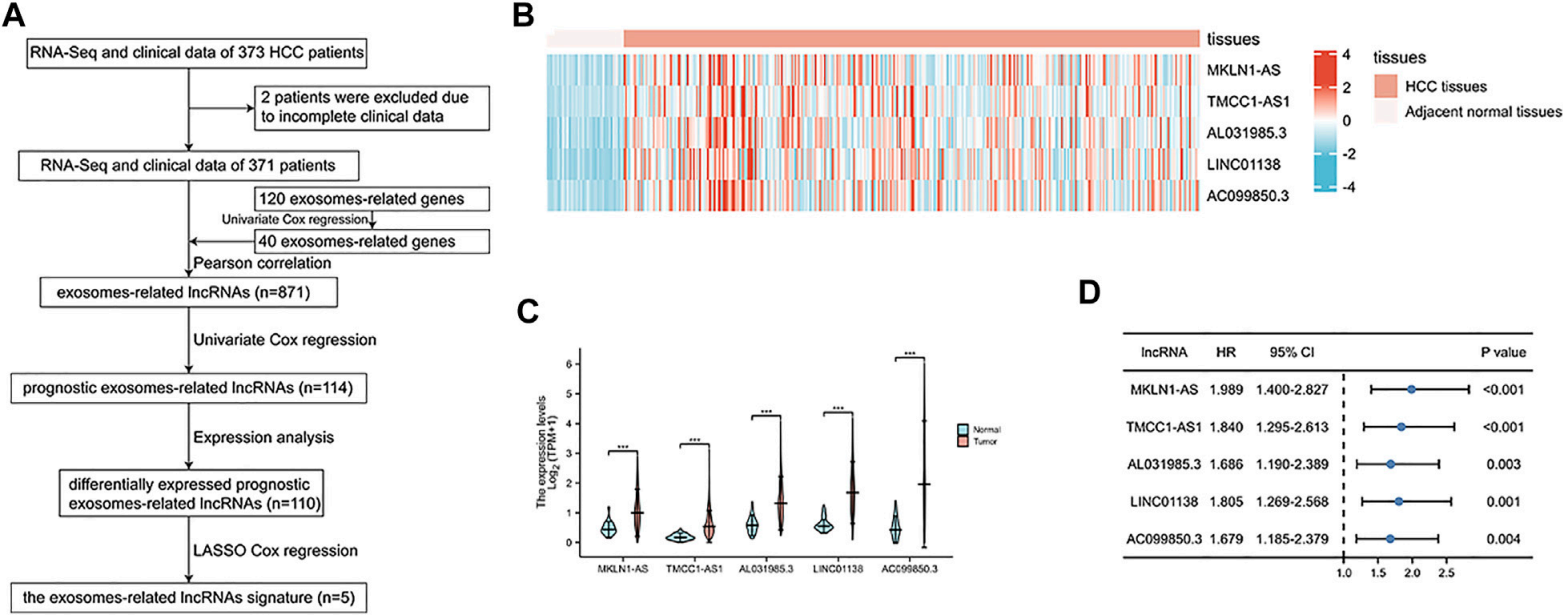
Identification of prognostic exosomes-associated lncRNAs in liver cancer patients. **(A)** The flow chart for the construction of exosome-related lncRNA signature. **(B)** Heatmap of 5 prognostic exosome-related lncRNAs in cancer tissues and adjacent normal tissues. **(C)** The expression of 5 prognostic exosome-related lncRNAs in normal and tumor tissues. **(D)** Univariate Cox regression of 5 prognostic exosome-related lncRNAs. *p < 0.05; **p < 0.01; ***p < 0.001; ****p < 0.0001.

**FIGURE 2:**
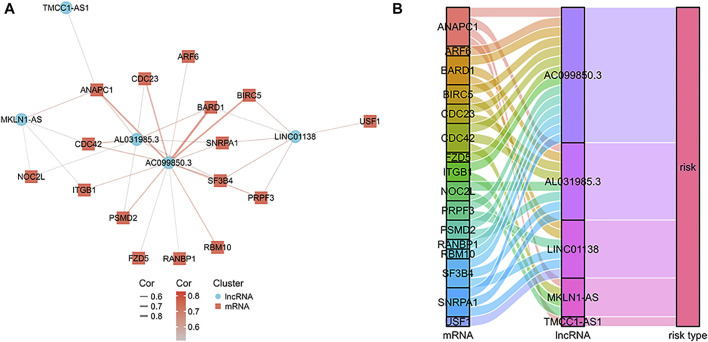
The mRNA-lncRNA co-expression network. **(A)** The co-expression network of the exosome-related genes and candidate lncRNAs. **(B)** Sankey diagram showing the connection degree between the exosome-related genes and candidate lncRNAs.

Based on previous studies, we obtained 120 exosome-related genes (Wang et al., 2021; Xu et al., 2022). We obtained 40 prognosis-related exosomal genes through univariate Cox regression analysis. After Pearson correlation analysis of RNA sequence information and prognosis-related exosomal genes from 371 patients, 871 exosomal lncRNAs were preliminarily obtained. Then, the roles of exosome-related lncRNAs in patients prognosis were identified with a Pearson correlation coefficient |R2| > 0.5. Through univariate Cox regression analysis, 114 lncRNAs involved in the prognosis of liver cancer patients were obtained. Among these lncRNAs, 110 exosomal lncRNAs were differentially expressed in liver cancer samples. LASSO regression analysis in the training cohort was used to reduce our candidate genes and build risk models. Finally, five prognostic-related exosomal lncRNAs were obtained by LASSO regression analysis in the training cohort. The research flow chart is shown in Figure 1A. The heatmap showed the upregulated expression of five candidate lncRNAs in liver cancer and surrounding normal tissues (Figure 1B). Similarly, Figure 1C shows the expression profiles of five lncRNAs in normal tissues and tumor tissues, and their expression was significantly different. Through univariate regression analysis, we identified these five lncRNAs as potential factors predictive of the outcomes of the patients (Figure 1D). The exosome-related genes and lncRNAs obtained by coexpression analysis are shown in Figure 2A. It is worth noting that AC099850.3 was associated with 15 exosome-related genes. In addition, the Sankey diagram illustrated the relationship between exosomal mRNAs, lncRNAs, and risk types (Figure 2B). All of these data support potential roles of the five candidate lncRNAs in liver cancer.

### Construction and verification of the exosomes-related LncRNA signature

**FIGURE 3:**
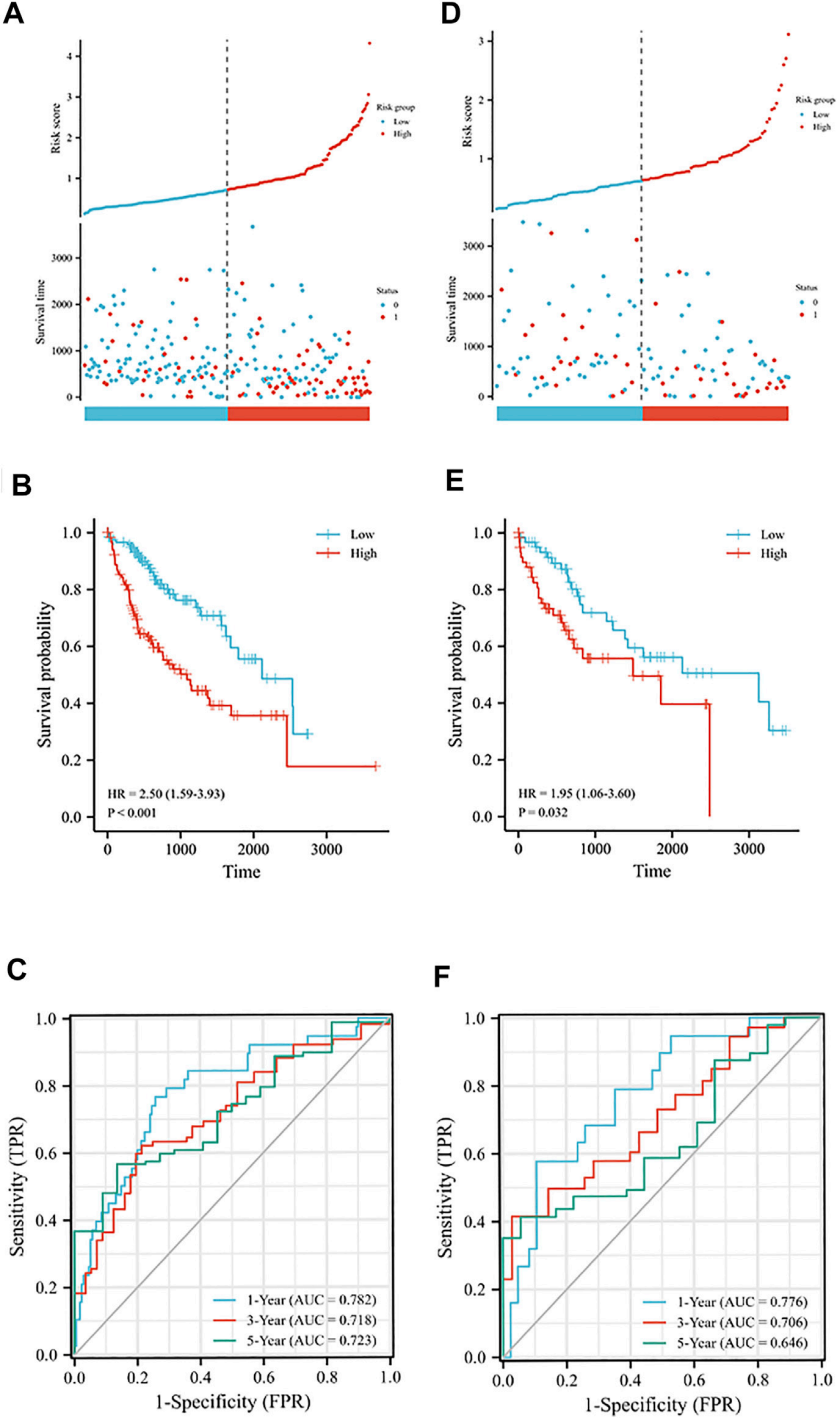
Prognostic analysis of exosome-related lncRNA signature in the training and validation cohorts. **(A)** Distribution of risk scores and overall survival status in the training cohort. **(B)** Kaplan-Meier curves for the overall survival of patients in the high- and low-risk groups in the training cohort. **(C)** The time-dependent ROC curves supporting prognostic accuracy of the risk score in the training cohort. **(D)** Distribution of risk scores and overall survival status in the validation cohort. **(E)** Kaplan-Meier curves for the overall survival of patients in the high- and low-risk groups in the validation cohort. **(F)** The time-dependent ROC curves supporting prognostic accuracy of the risk score in the validation cohort.

Using LASSO regression, we constructed a five exosomal lncRNA signature associated with the patient prognosis. We calculated our risk score according to the following formula: (0.19396×MKLN1-AS) + (0.20146×TMCC1-AS1) + (0.03562×AL031985.3) + (0.07893×LINC01138) + (0.0256×AC099850.3). To further verify the prognostic values of this risk model, we then divided all patients into high-risk and low-risk groups in the two cohorts according to their median risk score (Figure 3A,D). The signature and demographic characteristics of the two cohort samples are shown in Tables 2, 3. The survival analysis showed that the survival of high-risk patients in the two cohorts was worse than that of low-risk patients (Figure 3B,C). The ROC curve indicated that the area under the curve (AUC) reached 0.782 at 1 year, 0.718 at 3 years, and 0.723at 5 years in the training cohort and 0.776 at 1 year, 0.706 at 3 years, and 0.646 at 5 years in the validation cohort (Figure 3E,F). Subsequently, the multivariate and univariate regression analyses in the two cohorts suggested that this risk score could be an independent risk factor for the prognosis of liver cancer patients (Table 4, 5).

**TABLE 2:** Associations between the signature and patients’ characteristics in the training cohort.

Characteristic	Low-risk group (*n* = 124)	High-risk group (*n* = 123)	*p*-value
Age, median (IQR)	62 (54.75, 69)	61 (51, 68)	0.188
BMI, median (IQR)	24.5 (22.45, 28.67)	24.02 (21, 27.36)	0.069
Gender, n (%)			0.713
Female	41 (16.6%)	37 (15%)	
Male	83 (33.6%)	86 (34.8%)	
Family cancer history, n (%)			0.793
No	64 (30.5%)	72 (34.3%)	
Yes	37 (17.6%)	37 (17.6%)	
Race, n (%)			0.850
American indian or alaska native	1 (0.4%)	1 (0.4%)	
Asian	52 (21.6%)	53 (22%)	
Black or african American	4 (1.7%)	7 (2.9%)	
White	63 (26.1%)	60 (24.9%)	
Alcohol consumption, n (%)			1.000
No	75 (31.9%)	74 (31.5%)	
Yes	44 (18.7%)	42 (17.9%)	
Hepatitis B, n (%)			0.759
No	83 (35.3%)	84 (35.7%)	
Yes	36 (15.3%)	32 (13.6%)	
Hepatitis C, n (%)			0.634
No	100 (42.6%)	101 (43%)	
Yes	19 (8.1%)	15 (6.4%)	
T stage, n (%)			0.031
T1	69 (28.2%)	47 (19.2%)	
T2	26 (10.6%)	36 (14.7%)	
T3	24 (9.8%)	33 (13.5%)	
T4	3 (1.2%)	7 (2.9%)	
N stage, n (%)			0.662
N0	85 (34.6%)	84 (34.1%)	
N1	1 (0.4%)	3 (1.2%)	
NX	38 (15.4%)	35 (14.2%)	
M stage, n (%)			0.574
M0	91 (36.8%)	85 (34.4%)	
M1	2 (0.8%)	1 (0.4%)	
MX	31 (12.6%)	37 (15%)	
Pathologic stage, n (%)			0.024
Stage I	64 (27.7%)	44 (19%)	
Stage II	25 (10.8%)	31 (13.4%)	
Stage III	24 (10.4%)	39 (16.9%)	
Stage IV	3 (1.3%)	1 (0.4%)	
Neoplasm histologic grade, n (%)			0.039
G1	21 (8.6%)	15 (6.1%)	
G2	65 (26.6%)	48 (19.7%)	
G3	34 (13.9%)	52 (21.3%)	
G4	3 (1.2%)	6 (2.5%)	
Child-pugh classification grade, n (%)			0.546
A	83 (52.9%)	59 (37.6%)	
B	7 (4.5%)	8 (5.1%)	
Microvascular invasion, n (%)			0.005
Yes	28 (13.3%)	44 (21%)	
None	83 (39.5%)	55 (26.2%)	

**TABLE 3:** Associations between the signature and patients’ characteristics in the validation cohort.

Characteristic	Low-risk group (*n* = 62)	High-risk group (*n* = 62)	*p*-value
Age, median (IQR)	61 (48.25, 72.75)	59 (54, 68.75)	0.978
BMI, median (IQR)	25.35 (21.07, 31.01)	24.33 (21.03, 29.24)	0.458
Gender, n (%)			0.706
Female	20 (16.1%)	23 (18.5%)	
Male	42 (33.9%)	39 (31.5%)	
Family cancer history, n (%)			0.316
No	33 (30%)	39 (35.5%)	
Yes	22 (20%)	16 (14.5%)	
Race, n (%)			0.285
Asian	26 (21.7%)	27 (22.5%)	
Black or african American	1 (0.8%)	5 (4.2%)	
White	32 (26.7%)	29 (24.2%)	
Alcohol consumption, n (%)			0.372
No	46 (39.3%)	40 (34.2%)	
Yes	13 (11.1%)	18 (15.4%)	
Hepatitis B, n (%)			0.590
No	39 (33.3%)	42 (35.9%)	
Yes	20 (17.1%)	16 (13.7%)	
Hepatitis C, n (%)			0.089
No	52 (44.4%)	43 (36.8%)	
Yes	7 (6%)	15 (12.8%)	
T stage, n (%)			0.257
T1	34 (27.4%)	31 (25%)	
T2	13 (10.5%)	19 (15.3%)	
T3	11 (8.9%)	12 (9.7%)	
T4	3 (2.4%)	0 (0%)	
TX	1 (0.8%)	0 (0%)	
N stage, n (%)			1.000
N0	41 (33.1%)	42 (33.9%)	
NX	21 (16.9%)	20 (16.1%)	
M stage, n (%)			0.417
M0	47 (37.9%)	43 (34.7%)	
M1	1 (0.8%)	0 (0%)	
MX	14 (11.3%)	19 (15.3%)	
Pathologic stage, n (%)			0.697
Stage I	33 (28.4%)	30 (25.9%)	
Stage II	13 (11.2%)	17 (14.7%)	
Stage III	10 (8.6%)	12 (10.3%)	
Stage IV	1 (0.9%)	0 (0%)	
Neoplasm histologic grade, n (%)			0.001
G1	15 (12.3%)	4 (3.3%)	
G2	35 (28.7%)	29 (23.8%)	
G3	10 (8.2%)	26 (21.3%)	
G4	1 (0.8%)	2 (1.6%)	
Child-pugh classification grade, n (%)			0.417
A	40 (48.8%)	35 (42.7%)	
B	2 (2.4%)	4 (4.9%)	
C	1 (1.2%)	0 (0%)	
Microvascular invasion, n (%)			0.239
Yes	16 (15.2%)	21 (20%)	
None	39 (37.1%)	29 (27.6%)	

**TABLE 4:** Univariate and multivariate analyses of risk factors in the training cohort.

Variables	HR (95%CI)	*p*-value
Univariate analyses		
Age (years)	1.001 (0.984–1.019)	0.878
Gender (male vs. female)	0.915 (0.587–1.425)	0.694
BMI	0.967 (0.925–1.010)	0.126
Child-Pugh classification (B and C vs. A)	2.656 (1.165–6.052)	0.020
Alcohol consumption (Yes vs. No)	0.920 (0.579–1.463)	0.726
Hepatitis B (Yes vs. No)	0.303 (0.163–0.561)	<0.001
Hepatitis C (Yes vs. No)	0.929 (0.478–1.805)	0.827
Histologic grade (G3-4 vs. G1-2)	0.933 (0.596–1.460)	0.762
Microvascular invasion (Yes vs. No)	1.412 (0.831–2.401)	0.202
AJCC tumor stage (III and IV vs. I and II)	2.745 (1.744–4.320)	<0.001
Risk score	2.718 (2.089–3.538)	<0.001
Multivariate analyses		
Child-Pugh classification (B and C vs. A)	1.910 (0.805–4.534)	0.142
Hepatitis B (Yes vs. No)	0.420 (0.199–0.883)	0.022
AJCC tumor stage (III and IV vs. I and II)	1.669 (0.831–3.353)	0.150
Risk score	3.033 (1.762–5.220)	<0.001

**TABLE 5:** Univariate and multivariate analyses of risk factors in the validation cohort.

Variables	HR (95%CI)	*p*-value
Univariate analyses		
Age (years)	1.030 (1.007–1.053)	0.012
Gender (male vs. female)	0.642 (0.352–1.169)	0.147
BMI	1.035 (1.002–1.070)	0.038
Child-Pugh classification (B and C vs. A)	0.723 (0.169–3.085)	0.661
Alcohol consumption (Yes vs. No)	1.263 (0.651–2.449)	0.490
Hepatitis B (Yes vs. No)	0.468 (0.216–1.017)	0.055
Hepatitis C (Yes vs. No)	1.490 (0.703–3.155)	0.298
Histologic grade (G3-4 vs. G1-2)	1.576 (0.852–2.914)	0.147
Microvascular invasion (Yes vs. No)	1.294 (0.656–2.550)	0.457
AJCC tumor stage (III and IV vs. I and II)	1.961 (1.005–3.823)	0.048
Risk score	2.235 (1.378–3.627)	0.001
Multivariate analyses		
Age (years)	1.026 (0.997–1.057)	0.083
BMI	1.033 (0.999–1.067)	0.055
Hepatitis B (Yes vs. No)	1.102 (0.441–2.750)	0.836
AJCC tumor stage (III and IV vs. I and II)	2.135 (0.995–4.579)	0.051
Risk score	1.998 (1.065–3.751)	0.031

### Construction of a nomogram with clinicopathological information

**FIGURE 4:**
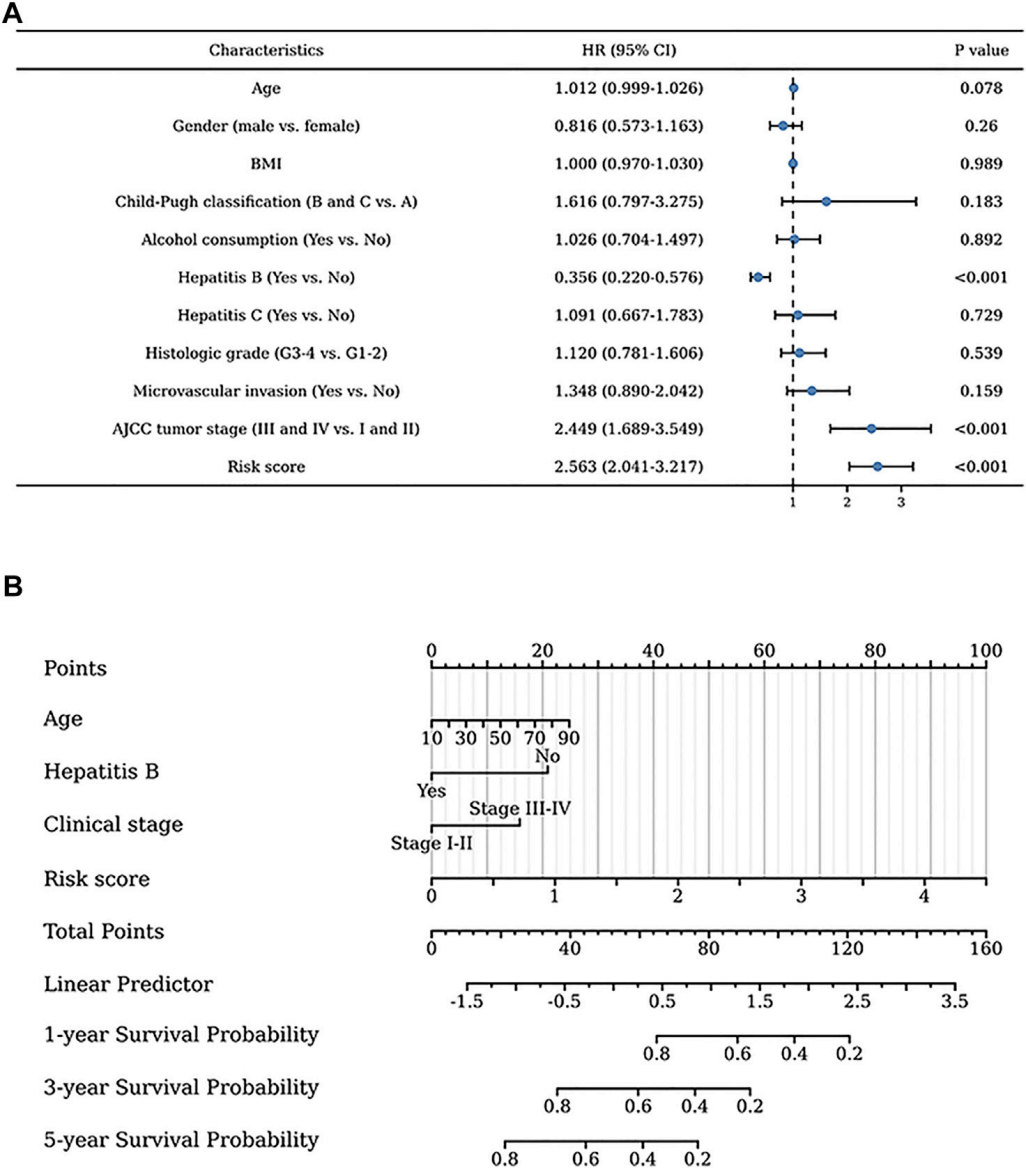
Clinical values of the exosome-related lncRNA signature. **(A)** The univariate regression analysis of clinical factors for the patients’ prognosis. **(B)** The nomogram was constructed to further identify the clinical applicability of this risk model.

Univariate regression analysis of all liver patients indicated that hepatitis B was a protective factor for the patients outcome, while the AJCC tumor stage and risk score were both risk factors for the outcome of HCC patients (Figure 4A). To further increase the clinical applicability of this model, a nomogram was constructed according to the multivariate regression analysis of patients’ prognostic factors. A nomogram including age, hepatitis B, clinical stage, and risk score was constructed to predict the 1-, 3-, and 5-years survival probability of liver patients with a C-index of 0.705 (Figure 4B).

### The relationship between the signature and immune-related pathways

**FIGURE 5:**
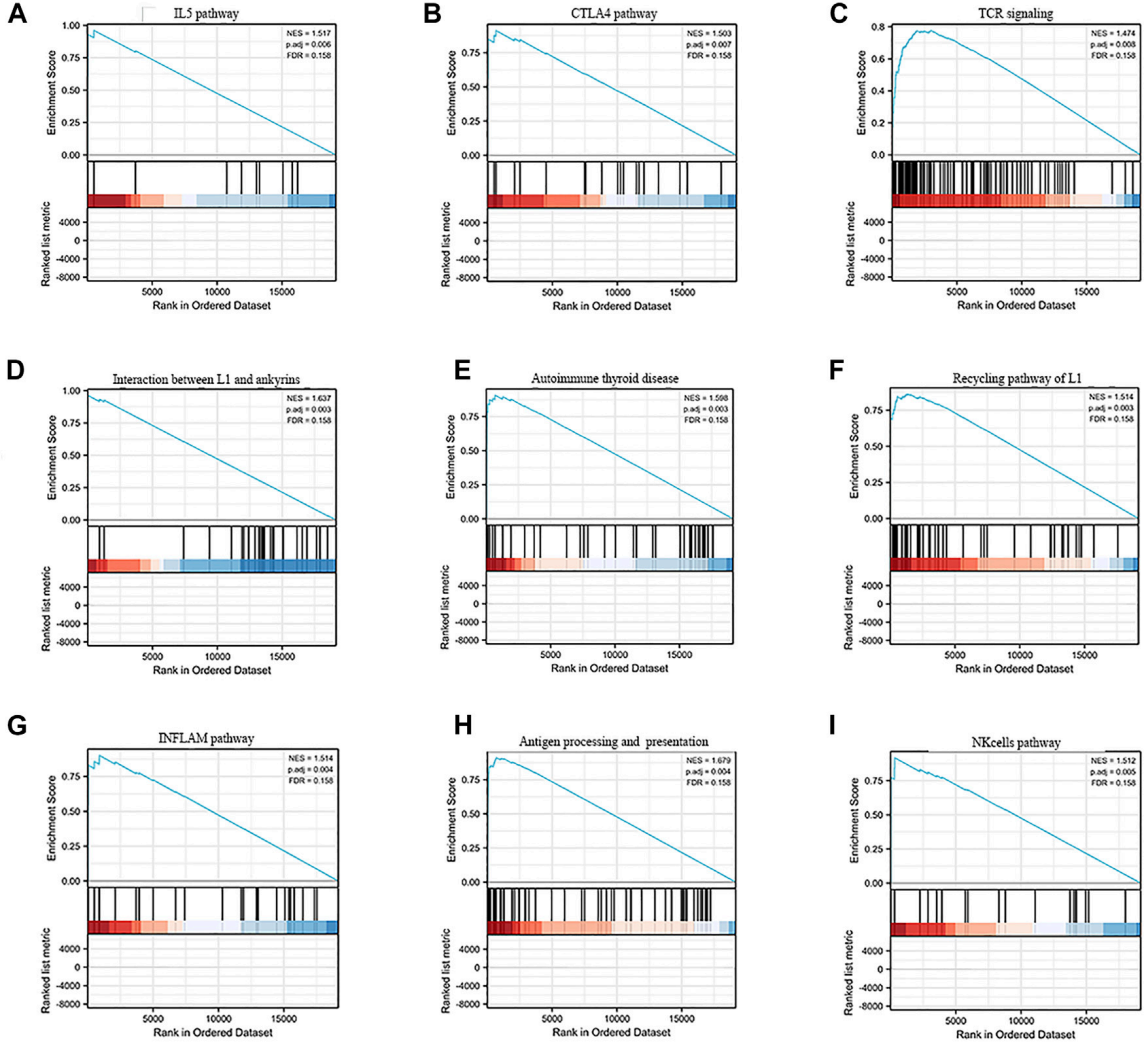
GSEA enrichment analysisof the exosome-related lncRNA prognostic signature. **(A–I)** The nine immune-associated signaling pathways regulated by this risk model, such as **(A)** IL5 pathway, **(B)** CTLA4 pathway, **(C)** TCR signaling, **(D)** Interaction between L1 and ankyrins, **(E)** Autoimmune thyroid disease, **(F)** Recycling pathway of L1, **(G)** INFLAM pathway, **(H)** Antigen processing and presentation and **(I)** NK cells pathway.

Gene set enrichment analysis (GSEA) revealed the underlying mechanisms of this signature in liver cancer (Supplementary Table S2). Figures 5A–I shows the nine immune-associated signaling pathways regulated by this risk model, such as the interaction between L1 and ankyrins, autoimmune thyroid disease, the recycling pathway of L1, the INFLAM pathway, antigen processing and presentation, the NK-cells pathway, the IL5 pathway, the CTLA4 pathway and TCR signaling. These results suggested the potential functional roles of the exosomes-related lncRNA signature in the regulation of the immune response in liver cancer patients.

**FIGURE 6:**
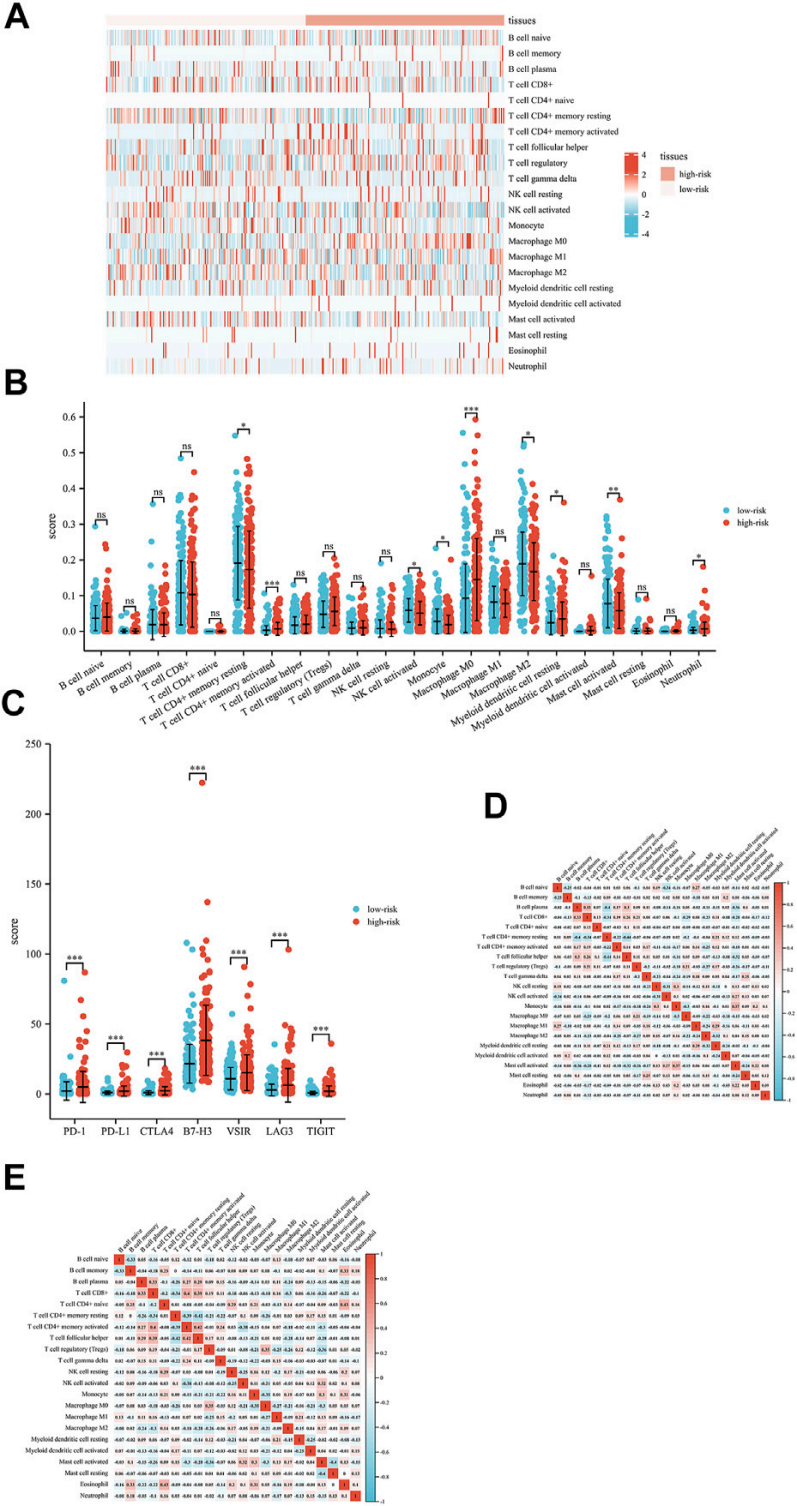
Interactions between the exosome-related lncRNA signature and immune regulation in liver cancer patients. **(A)** Heatmap of the tumor-infiltrating immune cell in low-risk and high-risk patients. **(B)** Comparisons of immune cells between the low-risk group and high-risk group. **(C)** Comparisons of multiple immune checkpoints between the low-risk group and high-risk group, including PD-1, PD-L1, CTLA4, B7-H3, VSIR, LAG3 and TIGIT. **(D)** Correlation matrix of immune cells in high-risk group patients. **(E)** Correlation matrix of immune cells in low-risk group patients. *p < 0.05; **p < 0.01; ***p < 0.001; ****p < 0.0001.

To better explore the relationship between this signature and the immune microenvironment, we used the CIBERSORT algorithm to evaluate the potential function of this signature in immune cell infiltration in liver patients. The distribution profiles of the tumor-infiltrating immune cells in liver cancer patients are shown in Figure 6A,B. T-cell CD4^+^ memory activation, M0 macrophages, resting myeloid dendritic cells, and neutrophils were highly expressed in patients with high risk scores. In addition, we explored the differential expression of several immune checkpoints in high-risk and low-risk HCC patients and found that Programmed death-1 (PD-1), Programmed cell death-ligand 1(PD-L1), Cytotoxic T-lymphocyte-associated protein 4 (CTLA4), B7 homolog 3 protein (B7-H3), V-Set Immunoregulatory Receptor (VSIR), Lymphocyte activation gene 3 (LAG3) and T-cell immunoreceptor with immunoglobulin and ITIM (TIGIT) were all highly expressed in high-risk patients (Figure 6C). Finally, the correlation analysis of different immune cells is shown in Figure 6D,E. These results might provide possible theoretical support for patients’ choice of immunotherapy methods and immunotherapy targets in the future.

## Discussion

At present, an increasing number of studies have shown that exosomes play a key role in the growth, metastasis, and drug resistance of various cancer cells (Cheng et al., 2019; Mai et al., 2021). Our study screened exosome-related lncRNAs related to prognosis and constructed a prognosis-related lncRNA risk model for liver cancer patients. We also constructed a nomogram combined with the patient’s risk score to predict the prognosis and survival possibility at 1, 3, and 5 years, which further increased the practicability of this model. Then, we analyzed GSEA and the immune microenvironment, which may reveal new targets for liver cancer treatment in the future. This study might provide a new perspective on risk stratification and immunotherapy in patients with HCC.

Exosome-associated RNAs have a unique expression profile reflecting tumor characteristics, and their role in tumor development and metastasis is gradually emerging (Liang et al., 2020; Shang et al., 2020). In recent years, studies have shown that an imbalance of exosomal lncRNAs participates in many pathological processes, including liver cancer (Verduci et al., 2019). However, the detailed effect of exosome-lncRNA interactions on the development and treatment of liver cancer is still unknown. The serum exosomal lncRNA FAL1 could significantly improve liver cancer cell proliferation and migration ability, proving that exosome-related lncRNA FAL1 is a novel diagnostic and therapeutic target for liver cancer (Li et al., 2018).

In our study, five upregulated exosome-related lncRNAs (MKLN1-AS, TMCC1-AS1, AL031985.3, LINC01138, and AC099850.3) were used to construct a prognosis-related risk model for liver cancer. To date, the lncRNA muskelin 1 antisense RNA (MKLN1-AS) has been proven to be a carcinogenic regulator in HCC, able to promote the growth of tumors (Pan et al., 2022). Other studies have shown that MKLN1-AS is regulated by SOX9 transcription and enhances the effect of SOX9 on the proliferation and epithelial-mesenchymal transition (EMT) of liver cancer cells (Guo et al., 2022). TMCC1-AS1 has been considered another oncogene in recent studies, and can promote the proliferation and migration of liver cancer cells (Chen et al., 2021). Genome-wide analysis of long noncoding RNAs in liver cancer indicated that TMCC1-AS1 was negatively associated with overall survival and recurrence-free survival (Cui et al., 2017).At the same time, some studies also found that TMCC1-AS1 can predict the response of HCC patients to chemotherapy and immunotherapy (Deng et al., 2020). In previous studies, lncRNA AL031985.3 has been shown to be related to the tumor microenvironment and the prognosis of liver cancer (Wu et al., 2021). LINC01138 promotes malignant behaviors by activating arginine methyltransferase 5 in liver cancer cells, revealing LINC01138/PRMT5 axis as an ideal target for liver cancer treatment (Lin et al., 2018). Many studies have found that lncRNA AC099850.3 can promote the malignancy degree of liver cancer (Zhong et al., 2022).

Previous studies have reported that the infiltration of T cells and B cells around tumors can improve the survival rate of liver cancer patients (Garnelo et al., 2017). Other studies have found that the infiltration of macrophages is related to a good prognosis in liver cancer patients. The lower infiltration of immune cells in hepatocellular carcinoma is related to the presence of neutrophils, NK cells, and resting mast cells (Rohr-Udilova et al., 2018). In our study, T-cell CD4^+^ memory activation, M0 macrophages, resting myeloid dendritic cells, and neutrophils are highly invasive in patients with high risk scores. These results suggested that the five-lncRNA signature might have an important impact on immune cell infiltration.

Due to the high degree of malignancy and postoperative recurrence rate of liver cancer, the five-year survival rate is still low (Peng et al., 2021; Yan et al., 2021). In recent years, the emergence of sorafenib and PD-1 inhibitors has greatly prolonged the survival of HCC patients. Our study found that liver cancer patients in the high-risk group had high expression of some immune checkpoints (PD-1, PD-L1, CTLA4, B7-H3, VSIA, LAG3 and TIGIT). In addition, this signature was significantly related to the tumor immune microenvironment, providing potential new immunotherapeutic targets for patients with liver cancer. Moreover, our signature also comprehensively evaluated the prognostic values of liver cancer patients by combining five exosomal lncRNAs, which might help clinicians better manage patients and choose more appropriate treatment methods.

## Conclusion

In conclusion, we constructed a signature composed of five exosomal lncRNAs and proved the predictive value of this risk model for the outcome of liver cancer patients. This model is closely related to the immune cell microenvironment and provides a potential direction for research on liver cancer immunotherapy.

## Data Availability

The original contributions presented in the study are included in the article/Supplementary Material, further inquiries can be directed to the corresponding authors.
